# Supplementary material for: Treatment of Basal Cell Carcinoma with Electrochemotherapy: Insights from the InspECT Registry (2008–2019)
Source: Curr Oncol. 2022 Jul 28;29(8):5324–37. doi: 10.3390/curroncol29080423 (PMC9406883; doi:10.3390/curroncol29080423)
Supplement: Supplementary file 1 [file curroncol-29-00423-s001.zip › curroncol-1823365-supplementary.pdf]

Current Oncology

Article

Treatment of Basal Cell Carcinoma with Electrochemotherapy: Insights from the InspECT Registry (2008–2019)

Giulia Bertino, Tobian Muir, Joy Odili, Ales Groselj, Roberto Marconato, Pietro Curatolo, Erika Kis, Camilla Kjaer Lonkvist, James Clover, Pietro Quaglino, Christian Kunte, Romina Spina, Veronica Seccia, Francesca de Terlizzi, Luca Giovanni Campana and the InspECT BCC Working Group

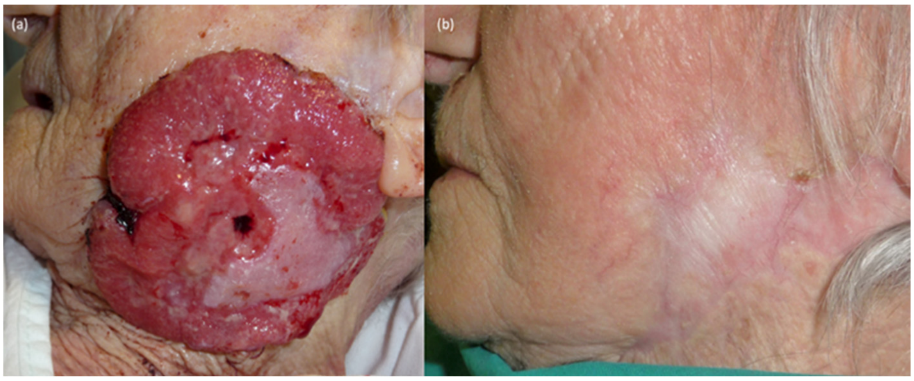

Figure S1. Primary locally advanced BCC in a 96-year-old patient treated with ECT under general anaesthesia. (**a**) Baseline presentation. (**b**) Complete response at 11 months, following a single course of treatment.

Table S1. Predictors of objective response.

**Characteristics**	**Univariate analysis**			**Multivariate analysis**		
	**RR**	**C.I. 95%**	***P***	**RR**	**C.I. 95%**	***P***
Anatomical location (head & neck vs other)	5.92	1.77-19.79	0.004	4.96	1.22-20.06	**0.025**
BLM administration (i.v. vs i.t.)	0.89	0.28-2.88	0.850			
Previous irradiation (yes vs no)	0.09	0.02-0.38	0.001	0.20	0.03-1.13	0.069
Lymphedema (yes vs no)	3077	0-+∞	0.967			
Tumour size (≤ 30 mm vs > 30 mm)	6.14	1.83-20.60	0.003	4.77	1.20-18.98	**0.027**
Coverage of deep tumour margin (yes vs no)	3.50	1.00-12.22	0.049	0.95	0.12-7.43	0.959
Coverage of lateral tumour margins (yes vs no)	4.30	1.22-15.13	0.023	2.60	0.31-21.63	0.376
Presentation (recurrent vs primary)	0.54	0.14-2.08	0.371			
Previous treatments	0.12	0.03-0.57	0.007	0.17	0.03-0.85	**0.032**

(yes vs no)

Table S2. Patient characteristics according to development or not of local recurrence.

Characteristic	No recurrence (*n* = 272 patients with 534 tumours)	Local recurrence (*n* = 28 patients with 35 tumours)	*P*
Sex			
Males	172 (63%)	16 (57%)	0.6676
Females	100 (37%)	12 (43%)	
Age (years)	76 (64-96)	72 (23-93)	0.1277
No tumours / patient	1 (1-7)	1 (1-7)	
Presentation			
Primary naïve	164 (60%)	17 (61%)	0.6862
Primary persistent	64 (24%)	8 (28%)	
Recurrent	44 (16%)	3 (11%)	
Tumour size (cm)	13 (5-350)	12 (5-40)	
≤3	483 (90%)	34 (97%)	0.2400
>3	51 (50%)	1 (3%)	0.3037
Tumour anatomic location			
Head & Neck	422 (79%)	31 (89%)	0.3863
Trunk	65 (12%)	2 (5.5%)	
Limbs	47 (9%)	2 (5.5%)	
Previous treatment			
None	164 (60%)	17 (61%)	
Surgical excision	97 (36%)	10 (36%)	0.9977
Other[a]	9 (3%)	1 (3%)	
Unknown	2 (1%)	0	
Anesthesia			
Local	178 (65%)	22 (79%)	0.2329
Local + sedation	94 (35%)	6 (21%)	
Route of BLM administration			
Intratumoural	125 (46%)	8 (29%)	0.1179
Intravenous	147 (54%)	20 (71%)	
Electrode geometry			
Row needle	337 (63%)	22 (63%)	
Hexagonal needle	134 (25%)	13 (37%)	0.1128
Plate	40 (8%)	0	
Combination	23 (4%)	0	

Table S3. Clinical studies on ECT in unselected cancer populations, including patients with BCC

Author	Year	Study	No pts	No of tumours	BCC subtype	BCC presentation	T size (mm)	Drug	Route	CR (%)	Re-ECT (%)	F-up (mos)	Recurrence
Clover[1]	2020	Registry	298	567	n.r.	n.r.	n.r.	BLM	i.v./i.t.	71[a]	n.a.	n.r.	n.r.
De Giorgi[2]	2020	Case series	4	4	n.r.	Primary: 3<br>Recurrent: 1	0.5-1	CDDP	i.t.	50	0	12	no
Rotunno[3]	2018	Case series	10	n.r.	n.r.	Primary / Recurrent	10 (5-190)[b]	BLM	i.v.[c]	83[d]	26[b]	5	n.r. for histotype
Montuori[4]	2016	Case series	11	15	n.r.	n.r.	n.r.	BLM	i.v. / i.t.	100	9[e]	12	no
Groselj[5]	2018	Prospective cohort<br>Retrospective case series	12<br>16	17<br>25	n.r.	Primary / recurrent	21 (6-80)<br>17 (4-50)	BLM	i.v.[f]<br>i.v.	100<br>96	n.r.	n.r.	n.r. for histotype
Bertino[6]	2016	Observational multicentric	34	34	n.r.	Primary: 20<br>Recurrent: 14	31/34 < 3 cm	BLM	i.v. / i.t.	91	3	6	1-yr DFS 89%<br>(4 recurrences)
Campana[7]	2016	Observational multicentric	24	n.r.	n.r.	Primary / recurrent	15 (10-30)	BLM	i.v. / i.t.	67[d]	24[b]	13.9	n.r. for histotype
Campana[8]	2014	Retrospective case series	9	9	n.r.	primary / recurrent	35 (10-100)	BLM	i.v. / i.t.	78[a, d]	39[b]	14	1-yr LPFS 86%
Rodriguez-Cuevas[9]	2001	Phase II	9	9	n.r.	primary: 8<br>recurrent: 1[g]	16±4	BLM	i.t.	78[a, d]	33	8.6	no

Note: BLM, bleomycin; CDDP, cisplatin; CR, clearance rate; i.t., intratumoural; i.v., intravenous; LDFS, local disease-free survival; LPFS, local progression-free survival; mos, months; n.e., not evaluable; n.r., not reported; SCC, squamous cell carcinoma. [a] per-tumour assessment; [b] also includes patients with other histotypes; [c] reduced BLM doses (13,500 / 10,000 / 7,500 IU/m^2); [d] per-patient assessment; [e] one patient received a second ECT for a newly diagnosed BCC outside the previous treatment field; [f] reduced BLM dose (10,000 IU/m^2); [g] after previous RT.

References

1. Clover, A.J.P.; de Terlizzi, F.; Bertino, G.; Curatolo, P.; Odili, J.; Campana, L.G.; Kunte, C.; Muir, T.; Brizio, M.; Sersa, G. Electrochemotherapy in the treatment of cutaneous malignancy: Outcomes and subgroup analysis from the cumulative results from the pan-European International Network for Sharing Practice in Electrochemotherapy database for 2482 lesions in 987 patients (2008–2019). *Eur. J. Cancer* **2020**, *138*, 30–40. https://doi.org/10.1016/j.ejca.2020.06.020.

2. De Giorgi, V.; Scarfì, F.; Saqer, E.; Gori, A.; Tomassini, G.M.; Covarelli, P. The use of cisplatin electrochemotherapy in nonmelanoma skin cancers: A single-center study. *Dermatol. Ther.* **2020**, *33*, e13547.
3. Rotunno, R.; Campana, L.G.; Quaglino, P.; de Terlizzi, F.; Kunte, C.; Odili, J.; Gehl, J.; Ribero, S.; Liew, S.H.; Marconato, R.; et al. Electrochemotherapy of unresectable cutaneous tumours with reduced dosages of intravenous bleomycin: Analysis of 57 patients from the International Network for Sharing Practices of Electrochemotherapy registry. *J. Eur. Acad. Dermatol. Venereol.* **2018**, *32*, 1147–1154. https://doi.org/10.1111/jdv.14708.
4. Montuori, M.; Santurro, L.; Feliziani, A.; Ricciardi, E.; Gaudio, D.; Campione, E.; Bianchi, L.; Silvi, M.B; Rossi, P. Electrochemotherapy for basocellular and squamocellular head and neck cancer: preliminary experience in Day Surgery Unit. *G. Ital. Dermatol. Venereol.* **2016**, *153*, 19–25.
5. Groselj, A.; Bosnjak, M.; Strojan, P.; Krzan, M.; Cemazar, M.; Sersa, G. 2018. Efficiency of electrochemotherapy with reduced bleomycin dose in the treatment of nonmelanoma head and neck skin cancer: Preliminary results. *Head Neck* **2018**, *40*, 120–125.
6. Bertino, G.; Sersa, G.; de Terlizzi, F.; Occhini, A.; Plaschke, C.C.; Groselj, A.; Langdon, C.; Grau, J.J.; McCaul, J.A.; Heuveling, D.; et al. European Research on Electrochemotherapy in Head and Neck Cancer (EURECA) project: Results of the treatment of skin cancer. *Eur. J. Cancer* **2016**, *63*, 41–52. https://doi.org/10.1016/j.ejca.2016.05.001.
7. Campana, L.G.; Testori, A.; Curatolo, P.; Quaglino, P.; Mocellin, S.; Framarini, M.; Borgognoni, L.; Ascierto, P.A.; Mozzillo, N.; Guida, M. Treatment efficacy with electrochemotherapy: A multi-institutional prospective observational study on 376 patients with superficial tumours. *Eur. J. Surg. Oncol.* **2016**, *42*, 1914–1923. https://doi.org/10.1016/j.ejso.2016.06.399.
8. Campana, L.G.; Mali, B.; Sersa, G.; Valpione, S.; Giorgi, C.A.; Strojan, P.; Miklavcic, D.; Rossi, C.R. Electrochemotherapy in nonmelanoma head and neck cancers: A retrospective analysis of the treated cases. *Br. J. Oral Maxillofac. Surg.* **2014**, *52*, 957–964. https://doi.org/10.1016/j.bjoms.2014.08.004.
9. Rodríguez-Cuevas, S.; Barroso-Bravo, S.; Almanza-Estrada, J.; Cristóbal-Martínez, L.; González-Rodríguez, E.; Electrochemotherapy in primary and metastatic skin tumors: phase II trial using intralesional bleomycin. *Arch. Med. Res.* **2001**, *32*, 273–276.
